# Highly resolved projections of climate and vegetation indicate major changes in northern Scandinavia: Implications for reindeer herding, tourism and nature conservation

**DOI:** 10.1007/s13280-026-02423-w

**Published:** 2026-05-22

**Authors:** Håkan Pleijel, Fredrik Lagergren, Erik Kjellström, Petter Lind, Paul A. Miller, Robert G. Björk, Tinja Olenius, Danijel Belušić, Mats P. Björkman, David Lindstedt, Gunhild Rosqvist, Camilla Andersson

**Affiliations:** 1 Department of Biological and Environmental Sciences, University of Gothenburg, P.O. Box 463, 40530 Göteborg, Sweden; 2 Department of Earth and Environmental Sciences, Lund University, Sölvegatan 12, 22362 Lund, Sweden; 3 Swedish Meteorological and Hydrological Institute, SMHI, 60176 Norrköping, Sweden; 4 Department of Earth Sciences, University of Gothenburg, P.O. Box 460, 40530 Göteborg, Sweden; 5 Department of Geophysics, Faculty of Science, University of Zagreb, Horvatovac 95, 10000 Zagreb, Croatia; 6 Department of Physical Geography, Stockholm University, 10691 Stockholm, Sweden

**Keywords:** Forest line, Rain-on-snow, Snow cover, Spatial resolution, Tree line, Zero crossing

## Abstract

Through Arctic amplification, global climate change is projected to disproportionally affect high-latitude environments. Additionally, the geographical heterogeneity and complex terrain of mountains promote climate-driven ecological change on fine spatial scales. We used a high-resolution convection-permitting regional climate model (HCLIM) and a state-of-the-art dynamic vegetation model (LPJ-GUESS) to explore mid-twentieth century implications of continued global warming for reindeer herding, tourism and nature conservation in the mountainous, northern parts of Scandinavia. Several indicators relevant for stakeholders were investigated: length of snow season, crossings of zero temperature, rain-on-snow events, as well as tree and forest line shifts. Comparing results from the high-resolution (3 km) climate model with results from a coarser-scale (12 km) model commonly used for climate change assessments revealed that ecologically and societally significant patterns become apparent only at higher resolution. Large changes are projected among all investigated indicators for the present century, and we explore their societal implications.

**Supplementary Information:**

The online version contains supplementary material available at https://doi.org/10.1007/s13280-026-02423-w.

## Introduction

Ongoing man-made climate change has become an established fact (IPCC 2021) and has the potential to profoundly alter the ecosystems of the Earth in interaction with other environmental drivers, such as air pollution and land use. High-latitude environments are experiencing larger increases of near-surface air temperature than the global average (Rantanen et al. 2023), resulting from the so-called Arctic amplification (AA, Manabe and Stouffer 1980). Several factors contribute to AA, including radiative forcing, local/regional feedback mechanisms and changes in long-range poleward energy transport (reviewed by Previdi et al. 2021). An example of regional feedback mechanisms is the amplified radiative forcing caused by increased carbon dioxide (CO_2_) levels in cold and dry air compared to warm and humid air closer to the equator. Other important feedbacks include changing vertical temperature gradients in the atmosphere and decreasing albedo (reflectivity) due to reduced snow and ice cover on land and sea. Increased poleward moisture transport also contributes to AA, while especially at higher warming levels with strongly reduced equator-to-pole temperature gradients, decreasing sensible heat fluxes dampen it. In general, AA makes high-latitude environments particularly vulnerable to climate change. Indeed, significant changes in vegetation composition and characteristics have been observed in Arctic areas, including increased vegetation height, a higher occurrence of erect shrubs and forbs resulting in decreasing species richness and altered species composition of lichen and bryophyte communities (Elmendorf et al. 2012; Bjorkman et al. 2018; García Criado et al. 2025).

Mountain areas are particularly sensitive to ecological changes, making them a significant concern with respect to climate change (Adler et al. 2022). For example, using climate scenarios for Europe, projections indicate that woody vegetation will replace the mountain tundra during this century (Hickler et al. 2012; Lagergren et al. 2024). Thus, vegetation zones are rapidly shifting towards higher altitudes, with shrubs expanding and trees advancing into the tundra (Gustafson et al. 2021), leading to habitat loss and potential local extinction of tundra species (Elsen and Tingley 2015), since plant species, dependent on e.g. seed dispersal for migration, can struggle to move uphill in response to changing climate conditions while birds have been shown to shift to higher elevations (Couet et al. 2022). 

Previous studies have often relied on coarse-resolution climate projections and broad climate metrics such as annual or monthly mean temperature and total amount of precipitation (Elmendorf et al. 2012; Berard-Chenu et al. 2021; García Criado et al. 2025), which fail to capture the fine-scale processes that determine ecological responses and societal impacts in mountainous regions. This limits the ability to assess risks for sectors such as reindeer herding, tourism and nature conservation, where adaptation decisions depend on specific climate indicators, often in complex or mountainous terrain, rather than general averages (Rutty et al. 2021; Adler et al. 2022; Rosqvist et al. 2022). For instance, winter tourism (Rutty et al. 2021; Rice et al. 2022) and reindeer herding (Harnesk 2022) are strongly dependent on a reliable and long-lasting snow cover and snow characteristics. Observations of reindeer behaviour suggest that weather and snow conditions significantly impact reindeer grazing opportunities (Rosqvist et al. 2022). Additionally, climate change and competing land use pose increasing challenges for reindeer to access good grazing grounds (Rosqvist et al. 2022). Studied indicators must therefore reflect a range of processes influencing ecosystems and livelihoods in areas like northern Scandinavia, such as changes in snow season length, zero temperature crossings and rain-on-snow events leading to ice formation, as well as shifts in tree and forest lines—variables that evidently strongly affect forage accessibility for reindeer, the viability of winter tourism and the persistence of tundra habitats (Hofgaard et al. 2012; Abegg et al. 2021; Adler et al. 2022; Rosqvist et al. 2022; Lagergren et al. 2024). By estimating these indicators with the help of high-resolution climate projections and dynamic vegetation modelling, it becomes possible to provide a more realistic basis for understanding ecological change and informing societal adaptation strategies.

To illustrate the relevance of these indicators, frequent temperature fluctuations crossing 0 °C increase the likelihood of rain-on-snow events, forming ice layers on the ground and in the snowpack, preventing reindeer from grazing during the snow season (Rasmus et al. 2022; Rosqvist et al. 2022). The need for adaptation of forestry to climate change is also an increasing concern of high importance in the region since climate change can increase the risk of windthrow, pests and droughts. In this context, forestry management systems, their intensity and the role of maintaining natural forests are critical for climate change adaptation (Keskitalo et al. 2016). For tourism, the shortening of the snow season, along with costs and efforts associated with artificial snow production to compensate for this, is of critical importance (Berard-Chenu et al. 2021; Rice et al. 2022). Furthermore, shifts in vegetation zones and altered vegetation composition resulting from climate change significantly affect the protection of biological diversity and other nature conservation values (Adler et al. 2022; Lagergren et al. 2024). Although no sharp boundaries exist between ecosystems, the transition zone from forest to treeless tundra (the forest–tundra–ecotone; e.g. Hofgaard et al. 2012) operationally represents changes in the forest line (the edge of the ecosystem below which trees form a closed canopy) and the tree line (the highest altitude or latitude where trees taller than 2 m grow depending on the environmental conditions). Especially, the advancement of the forest line, together with shrubification, leads to a loss of tundra environments and drastically alters the landscape character (Vowles and Björk 2019), affecting tourism and reindeer herding quantitatively and qualitatively. It also impacts nature conservation and changes conditions for forestry.

Improved projections of climate change impacts on high-latitude mountain ecosystems, such as the Scandinavian Mountain Range, require combining state-of-the-art climate projections (Jacob et al. 2020; Lucas-Picher 2021) with state-of-the-art dynamic vegetation modelling. Considering the high relief topographic heterogeneity of mountain areas, it is particularly important to apply a high spatial resolution in the modelling. Kilometre-scale (frequently called “convection-permitting”) climate models perform better than more simplified, coarser-scale models with respect to precipitation distribution in time and space (Lind et al. 2020). However, such models do not typically have a dynamic vegetation module, but rather use a land surface description with a fixed, present-day vegetation distribution when calculating the surface energy balance. To improve on this, we combined results from the HCLIM (HARMONIE-Climate) regional climate model (Belušić et al. 2020), with the LPJ-GUESS (The Lund-Potsdam-Jena General Ecosystem Simulator; Smith et al. 2001, 2014) dynamic vegetation modelling (DVM) simulations, using nitrogen deposition input from the chemistry transport model MATCH (Multi-scale Atmospheric Transport and CHemistry; Andersson et al. 2007). Importantly, LPJ-GUESS was applied with its recently developed reindeer grazing module (Lagergren et al. 2024), which simulates browsing pressure on vegetation—a key factor influencing forest–tundra dynamics and the balance between climate-driven vegetation change and grazing impacts (Vowles and Björk 2019). This integrated approach provides novel km-scale and consistent projections of indicators identified as important to stakeholders across the aforementioned sectors in northern Scandinavia. Our aims are:

- To evaluate the benefits of a higher spatial modelling resolution to assess the spatial and temporal development of societally important impacts of continued global warming by the mid-twentieth century and beyond in a topographically and ecologically heterogeneous area in northern Scandinavia.
- To generate projections of ecologically and societally important climate change indicators, including snow cover, rain-on-snow events, zero temperature crossings and extent and character of forest cover for the study area.
- To compare projections of temperature and precipitation in the study area for the middle and the end of the present century of two emission scenarios and discuss uncertainties in the modelling setup used.

## Geographical framing of the study

**Fig. 1:**
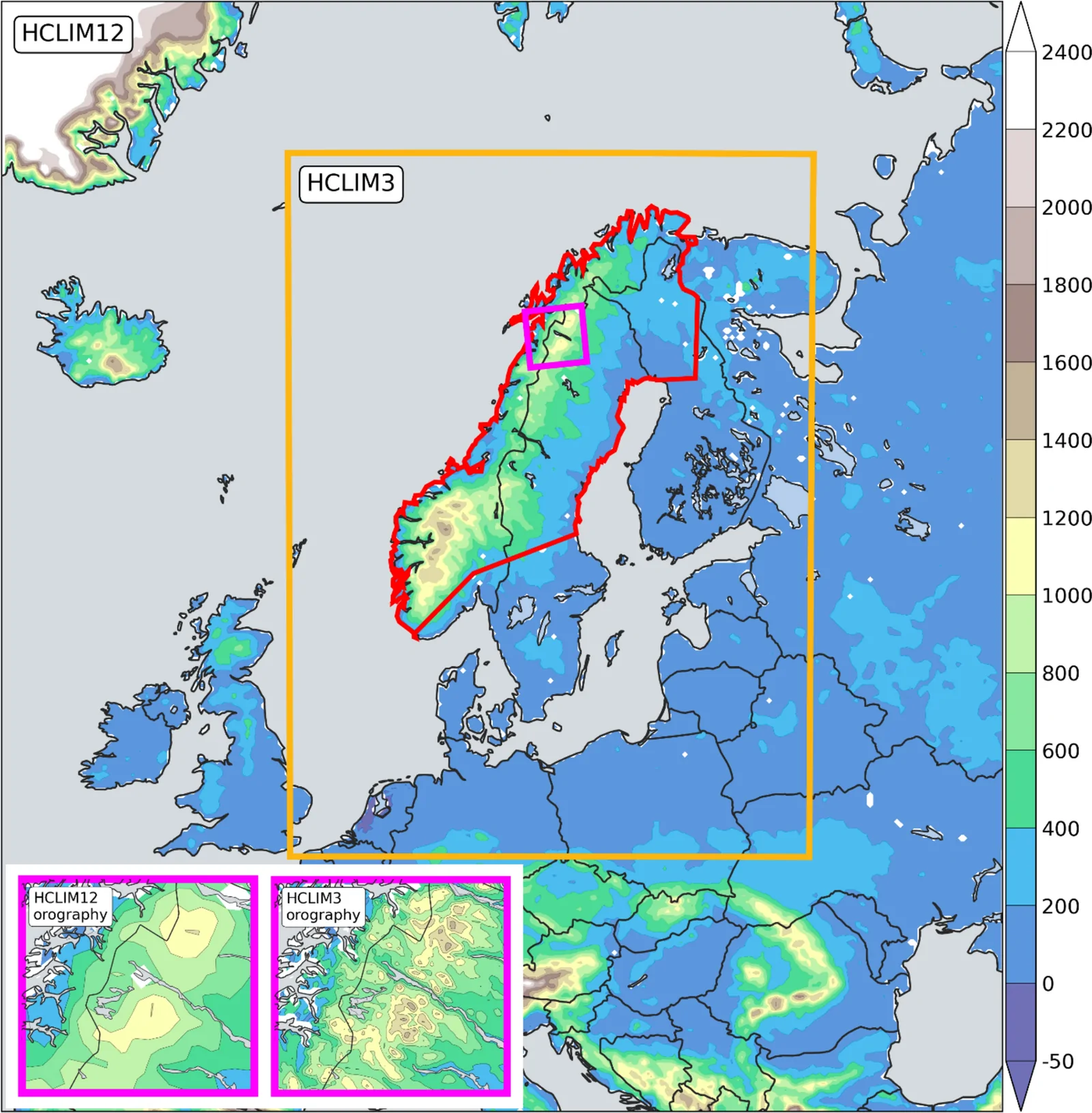
Areal extent of different aspects of the investigation in Northern Europe. For the climate modelling, the higher spatial resolution (3 km) of the climate simulation (HCLIM3, orange rectangle) was downscaled from a 12 km resolution simulation (HCLIM12, the entire map). To highlight the significance of the higher spatial resolution on snow cover, rain-on-snow events and zero temperature crossings, a smaller high-latitude area within the mountains was selected for analysis (magenta square). Insert maps at the bottom show the difference in model orography for this area for HCLIM12 and HCLIM3, respectively. For the dynamic vegetation modelling, the development of the vegetation composition, including the forest and tree lines, was modelled for the Fennoscandian boreal and mountain area (red borders). The colour scale to the right represents the orography of the model (m)

The study was conducted within the research programme Biodiv-Support^1^. Its focus was to investigate how ecosystems in high-latitude areas in Europe could be impacted by climate change and air pollution exposure. The geographical framing of this study is presented in Fig. 1. The area delineated by the red line in Fig. 1 contains the mountain range of Scandinavia and the adjacent boreal coniferous forest, predominantly located to the east of the mountains. The reindeer herding takes place within this area with seasonal movements between summer grazing areas in the mountains and the lowland forest areas in the winter. The modelling of the effects of climate change on vegetation composition was made for this area.

Three 90 × 90 km areas along a gradient in latitude with high nature conservation values were selected as hotspots for a more detailed analysis. From North to South, these were 1. Abisko (Northern Sámi: Ábeskovvu, 68°N, 19°E) which includes the Abisko National Park, known for its high plant and animal biodiversity and dramatic geological and natural geographical features, including the largest mountain lake in Scandinavia; it is also the site of the international Abisko Scientific Research Station, 2. Vindelfjäll (66°N, 16°E), the area containing the largest nature reserve in Sweden which is also one of the largest in Europe; the dramatic orography of the area extends from large mire areas and virgin spruce forests to high elevation mountain tundra, up to 1768 m a.s.l.; there is an ecological research station also in the Vindelfjäll area, 3. Helags (Southern Sámi: Maajåelkie, 63°N, 13°E) and the most elevated areas, reaching 1798 m a.s.l., in the southern part of the mountain range in Sweden also containing the southernmost glacier in this country. There is active reindeer herding in all three areas.

The magenta square in Fig. 1 contains some of the most elevated areas of the northern part of the Scandinavian Mountain range, presently with extensive areas with mountain tundra, but also extending to sea level areas in Norway. This area was used to investigate the effects of climate change on the stakeholder-relevant indicators snow cover, rain-on-snow events and zero temperature crossings and how the choice of geographical resolution affects the distribution of these climate change effects in the modelling. Analyses were conducted using two levels of geographical resolution (12 × 12 km^2^ and 3 × 3 km^2^) to evaluate how spatial detail influences modelled outcomes (Fig. 1). The implications of the geographical resolution for orography are shown by the insert to the lower left of Fig. 1.

## Materials and methods

The modelling setup of the study is summarized in Fig. S1 of the Supplementary information, with different model components described in detail below.

### Climate modelling

We used climate simulation data from the Nordic Convection-Permitting Climate Projections project (NorCP; Lind et al. 2020) with the HARMONIE-Climate (HCLIM) model cycle 38 (HCLIM38; Belušić et al. 2020) run with time steps of 75 and 300 s at 3 and 12 km horizontal grid spacing, respectively. To capture some scenario uncertainty in the simulated climate, HCLIM38 was used for downscaling two projections for the future with the global climate model (GCM) EC-Earth (Hazeleger et al. 2010, 2012), one under the representative concentration pathway (RCP) scenario RCP4.5 and one under RCP8.5 (Moss et al. 2010). To capture structural uncertainty arising from the climate model chosen, another projection with the GCM GFDL (Donner et al. 2011; Griffies et al. 2011) under RCP8.5 was also downscaled. All downscaling simulations were undertaken in two steps: first for a pan-European domain to 12 km with HCLIM38-ALADIN (HCLIM12 henceforth), and then further to 3 km for a domain covering the Baltic Sea and land areas surrounding it, including all of Fennoscandia (Fig. 1), with HCLIM38-AROME (HCLIM3 henceforth) for the periods 1985–2005, 2040–2060 and 2080–2100 (Lind et al. 2020, 2023). The first year has been considered as spin up in all analyses that consequently covers 20-year time windows. The only exception is the HCLIM12 simulation downscaling EC-Earth for RCP8.5, which was performed for the full period 1985–2100.

### Dynamic vegetation modelling

The vegetation development was simulated with LPJ-GUESS (v4.1, Smith et al. 2001, 2014; Gustafson et al. 2021) using a set of Arctic plant functional types (PFTs). The simulations applied a recently developed implementation of reindeer browsing and trampling (Lagergren et al. 2024), which reduces biomass of accessible leaves based on PFT-specific reindeer dietary preference and kills plant individuals based on PFT-specific trampling sensitivity. The model was driven by daily data from the EC-Earth RCP8.5 projection downscaled by HCLIM3 as described above for the 1985–2100 period, where the gaps from 2006–2039 and 2061–2079 in the HCLIM3 data were filled using the HCLIM12 data. For daily mean, minimum and maximum temperature a nonparametric empirical quantile mapping “QUANT” bias adjustment method (e.g. Osuch et al. 2017) was applied using observed 1 × 1 km data from the Nordic Gridded Climate Dataset (NGCD^2^) aggregated to the HCLIM3 grid (for further details see Lagergren et al. 2024, Section 2.3). The model output of leaf-area index (LAI) of tree PFTs was used to assess tree and forest line advancement. It should be noted that definitions of forest are typically based on a tree height above 5 m and canopy cover above a certain threshold, instead of LAI. Different tree canopy-cover thresholds for an area to be considered as forest have been applied, e.g. > 10% by FAO (2020), > 20% by Global Land Cover 2000 product of northern Eurasia as defined by Bartalev et al. (2003) and > 30% by Corine as defined by Kosztra et al. (2019). The relationship between canopy cover and LAI is not linear or direct (George et al. 2021; Li et al. 2022). In the present study, we used a threshold LAI of 0.5 m^2^ projected leaf area per m^2^ ground area to approximately define the forest line. This corresponds to 22% of light intercepted in the canopy applying the Beer–Lambert law assuming a light extinction coefficient of 0.5 (Monsi and Saeki 2005), which is more directly related to canopy coverage. The tree line is generally defined as the border of the habitat where trees survive. For example, Kullman (2016) used a practical definition of the altitude where the last tree with height above 2 m is found on a mountain slope. Here we set the threshold LAI value defining the tree line to 0.025 m^2^ m^−2^, roughly corresponding to 10–25 individual trees of 2–3 m height per 1000 m^2^.

Lagergren et al. (2024) compared the LPJ-GUESS simulations used here with satellite-derived landcover datasets for these regions and others and showed that the model agreed well with the satellite product. This reflects the quality of the model and the HCLIM3 driving data for the period.

### Air pollution modelling

Results from the Eulerian chemistry and transport model MATCH (Robertson et al. 1999; Andersson et al. 2007) were used to describe historical, current and future atmospheric nitrogen deposition for input to the dynamic vegetation model LPJ-GUESS. Historical deposition was obtained from the MATCH-ECLAIRE (Engardt et al. 2017) dataset available at 50 km resolution, whereas deposition from current to future was obtained from the MATCH-BIODIV dataset available at 12 km resolution (Andersson et al. 2025) for the period until 2051. After this, the LPJ-GUESS standard dataset (Lamarque et al. 2010) was used. Anthropogenic air pollutant emissions in MATCH-BIODIV, including NO_x_ and NH_3_, were obtained from the ECLIPSE V6b dataset following the current legislation scenario. For more details, see Lagergren et al. (2024).

## Results

To highlight aspects of the projected climate change for the present century, we contrast the periods 2041–2060 and 1986–2005. For projected vegetation change, we use three periods (1986–2005, 2041–2060 and 2081–2100). For the course of vegetation development, a continuous temporal scale for the full century is used.

### Duration of the snow cover season

**Fig. 2:**
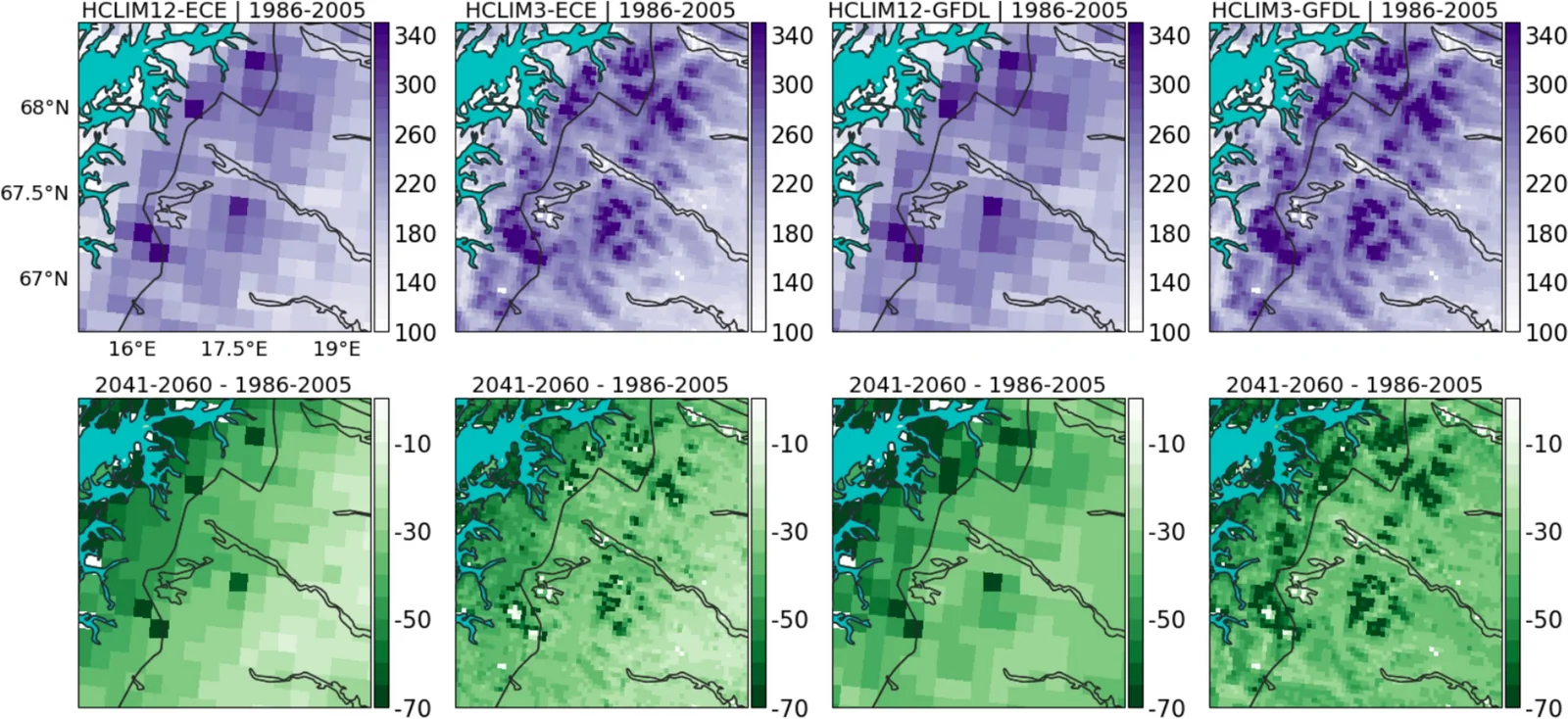
The upper four panels represent the number of days per year with a snow depth larger than 0.2 m during the historical period (1986–2005). The lower four panels show the difference in number days per year with a snow depth larger than 0.2 m between the mid-century (2041–2060) under the RCP8.5 scenario and the historical period. The four panels to the left show the results for the EC-Earth model, while the four panels to the right represent the GFDL model. The results for HCLIM12 (coarser spatial resolution, left panels) and HCLIM3 (higher spatial resolution, right panels) are compared. Latitudes and longitudes defining the area in northern Sweden and Norway are given as the y-axis and x-axis, respectively, in the upper left panel. Marine grid points in the models are marked light blue

We assumed that a snow depth of less than 0.2 m represents an incomplete snow cover with respect to, e.g. winter sports and other snow-dependent activities. The models projected substantial reductions in the duration of the snow cover by mid-century, especially in the westernmost parts of the study area (Fig. 2). This includes both areas close to the Atlantic coast, where the snow season is relatively short, and the highest-alpine areas of the mountains where the snow season is much longer. Comparison of the 3 km and the 12 km spatial resolution model results highlights that finer, local-scale aspects of the projected change only become apparent when using the higher resolution. The projections based on the two different GCMs (EC-Earth and GFDL) show very similar geographical patterns of change, but stronger effects in the case of the GFDL model, highlighting the uncertainty introduced by the driving GCM chosen for dynamical downscaling.

### Zero-crossings and rain-on-snow events

**Fig. 3:**
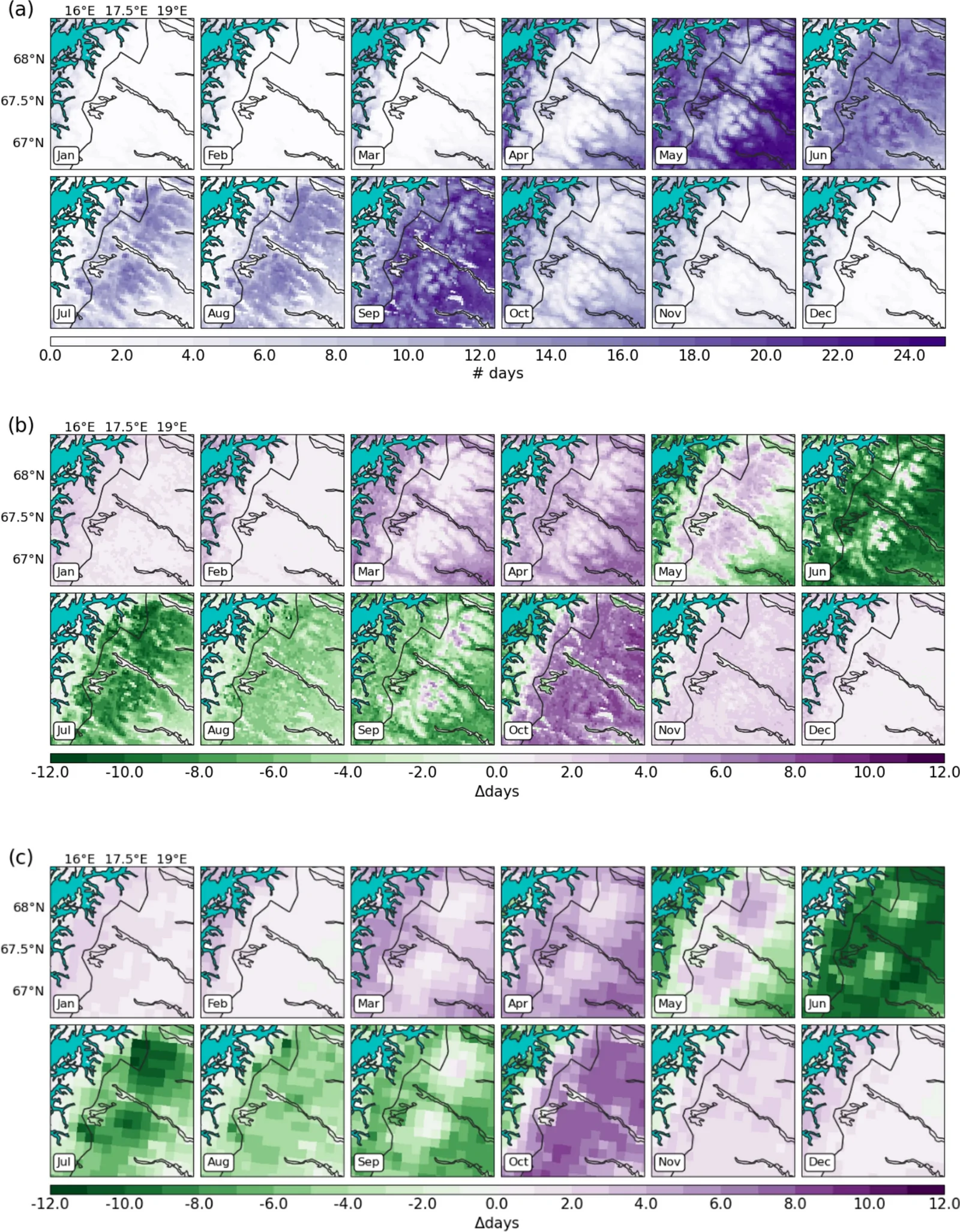
Number of days with zero-crossings (days when the temperature is crossing 0 °C at least once) for: **a** each month of the year in HCLIM3 downscaling EC-Earth for the historical period (1986–2005); the difference in number of days with zero-crossings between the middle of the century (2041–2060) under RCP8.5 and historical periods for **b** HCLIM3 (higher spatial resolution), and **c** HCLIM12 (coarser spatial resolution). Latitudes and longitudes defining the area in northern Sweden and Norway are given as the y-axis and x-axis, respectively, in the upper left panel. Marine grid points in the models are marked light blue

Zero-crossings (days when the temperature crosses 0 °C at least once) have historically been relatively rare during the period between October and April (Fig. 3a) but are projected to become substantially more common in the future climate of mid-century (Fig. 3b). Changes are most notable in the transition seasons between summer and winter, i.e. October to November and March to April and at high elevations in May. However, also in the colder winter months, December to February, the model projects an increasing number of zero-crossings, especially in the west, close to the Atlantic coast. From June to September, the results mostly show a clear decrease in zero-crossings as temperatures more prominently stay above 0 °C in this period. In June and September, exceptions are seen with increases in zero-crossings at higher elevations of the Sarek and Kebnekaise massifs. As expected, the fine pattern of geographical variation characteristic of mountain environments with complex topography is revealed to a much larger extent by the higher resolution of 3 km (HCLIM3; Fig. 3b) than by the coarser modelled spatial resolution of 12 km (HCLIM12; Fig. 3c).

**Fig. 4:**
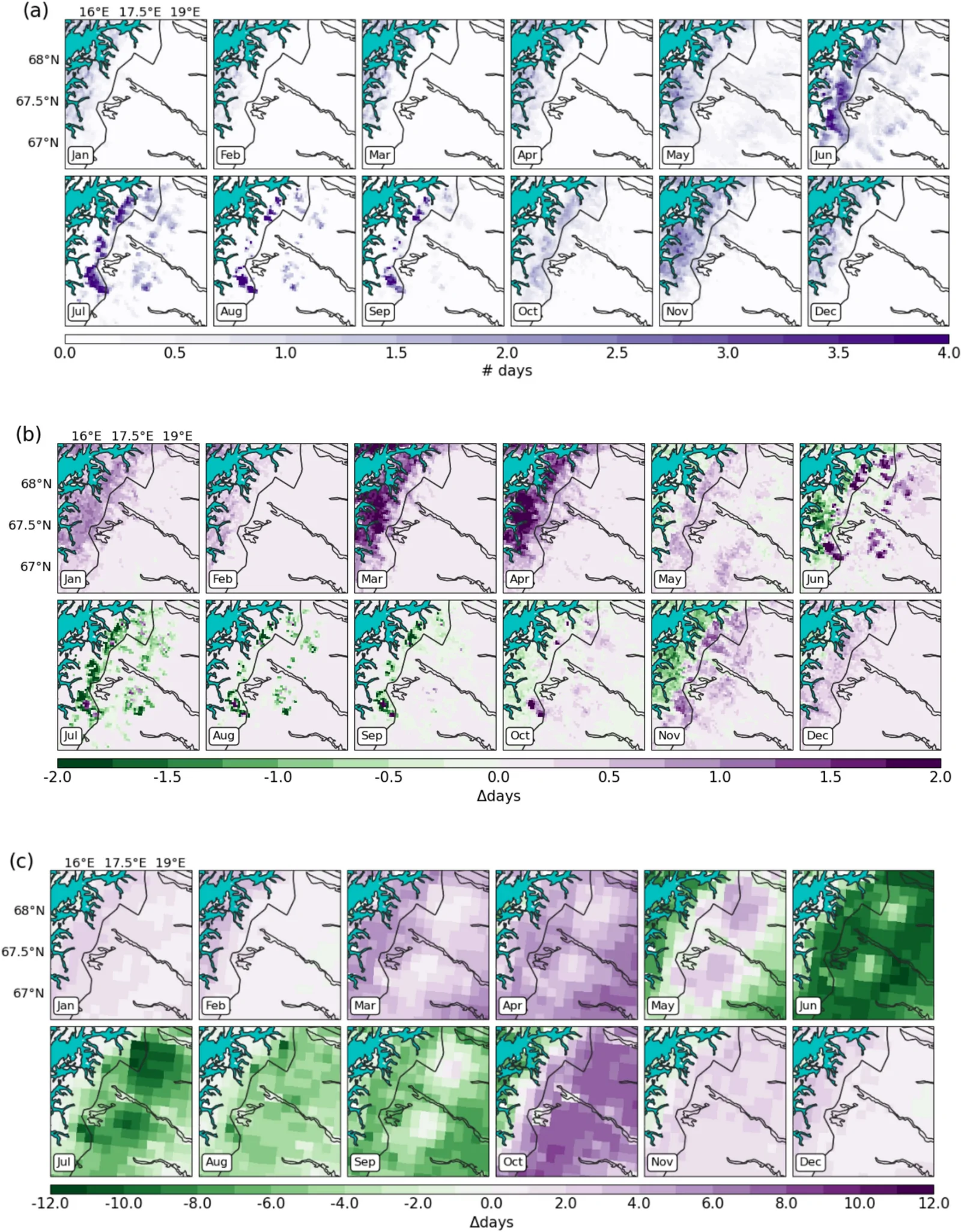
As Fig. 3 but showing the number of days with rain-on-snow events

**Fig. 5:**
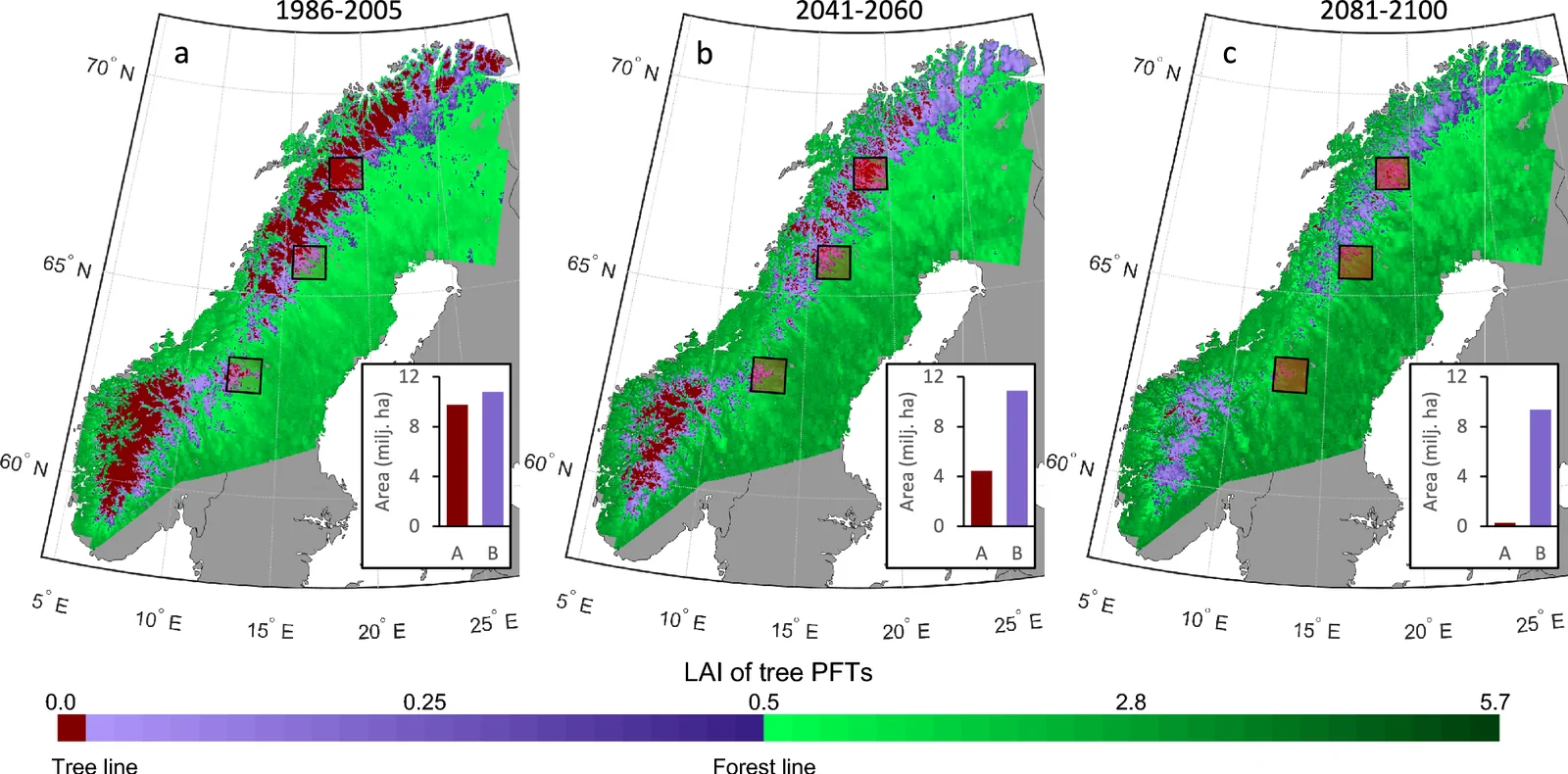
Simulated leaf-area index (LAI) of tree plant functional types averaged over the historical (1986–2005, **a**) near future (2041–2060, **b**) and late century (2081–2100, **c**) periods. The colour scale is adjusted to represent areas above the tree line with dark red colour, areas between the tree line (forest LAI = 0.025 m^2^ m^−2^) and the forest line (forest LAI = 0.5 m^2^ m^−2^) with purple shades and areas below the forest line with green shades. The total area above tree line (A) and between tree and forest line (B) are shown in the lower right inlets. The three shaded squares show the locations of the 90 × 90 km biodiversity hotspot areas. From North to South, these are Abisko (68°N, 19°E), Vindelfjäll (66°N, 16°E) and Helags (63°N, 13°E), which are shown in further detail in Fig. 6

The projected shorter snow season discussed above (Fig. 2) leads to fewer days with rain-on-snow (days with at least 10 mm rain that falls on snow cover and corresponds to at least 10 mm of snow water equivalents on the ground) in many areas during the period June to November (Fig. 4b). A decrease in number of rain-on-snow days is seen in June and October–November in regions close to the Atlantic, while more high-altitude areas are projected to have less rain-on-snow days in June–September. In the period November until May, the model results show a clear increase in the number of rain-on-snow events, especially in the areas closer to the Norwegian coast (from January to April) and in the high-altitude areas (from May to November, Fig. 4b). In June, October and November, these changes result in a complex geographical pattern including increases and decreases in the number of rain-on-snow days at different locations. Just as for zero-crossings, comparison of the HCLIM3 and HCLIM12 model results (Fig. 4b and 5c) emphasizes the benefits of using the higher spatial resolution, and consequently the uncertainty inherent in coarser resolution simulations of mountainous domains. Details are otherwise lost, such as the increase in rain-on-snow events in some areas in May, June and October.

### Vegetation dynamics—tree line and forest line

The latitudinal and altitudinal advance of the LAI boundaries, used to represent the tree line and the forest line, between the historical period (1986–2005) and the near future (2041–2060), is apparent (Fig. 5a, b). In this strong RCP 8.5 scenario, the areas above the tree line were projected to become very limited for the late century period 2081–2100 (Fig. 5c), which would be associated with a drastic change in the mountain landscape compared to the recent past. It can be noted that, although its geographical location shifts, the estimated area of sparser forest between the forest line and the tree line remained relatively constant within the simulated Scandinavian Mountain Range over the whole simulated period (Fig. 5). The area above the tree line was already by mid-century reduced by 55% (from 9.8 to 4.4 million ha) and only 2.7% (0.26 million ha) remained in the last period according to the model output.

**Fig. 6:**
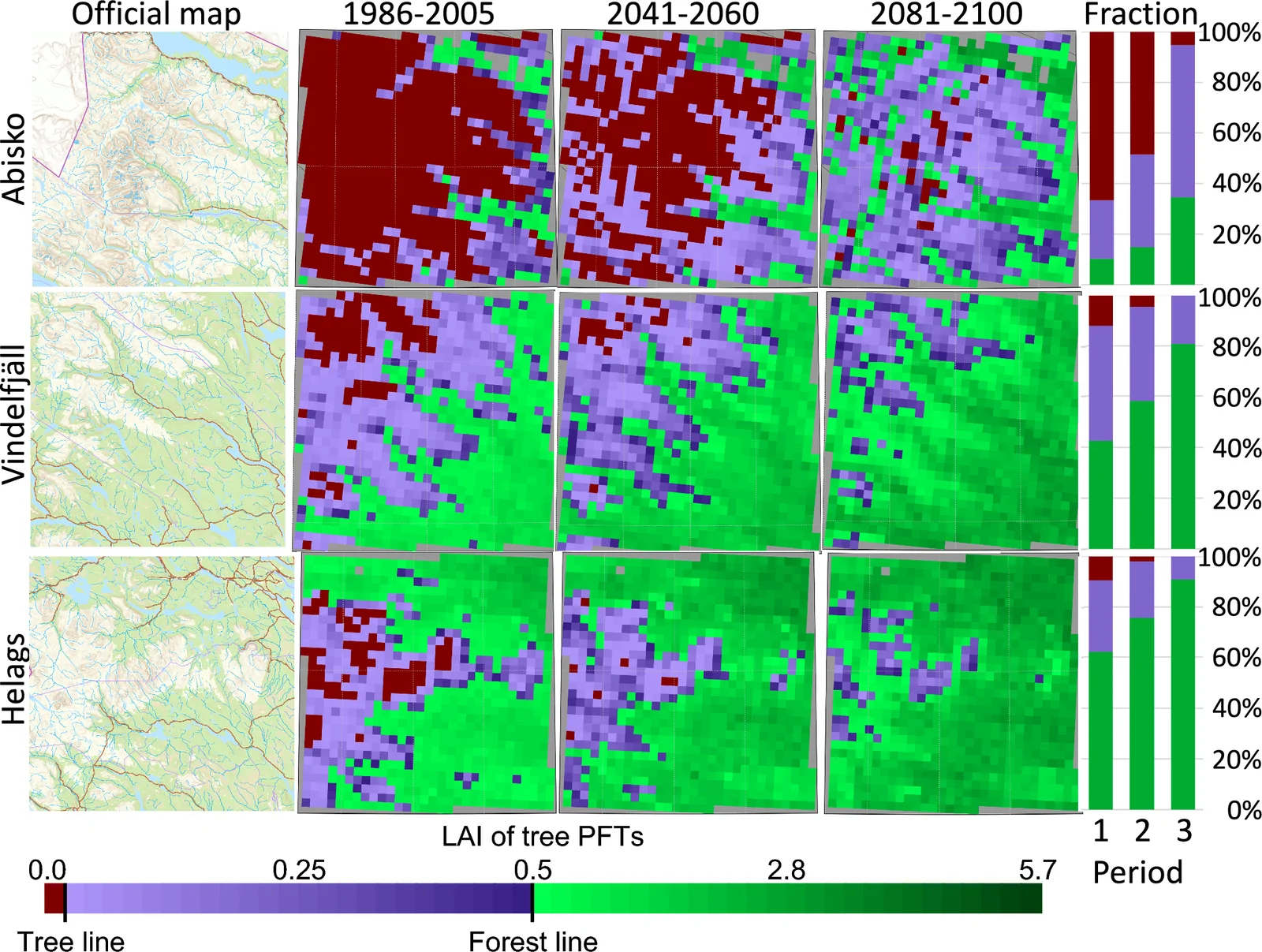
Illustration of changes in vegetation zones in three mountain biodiversity hotspot areas (90 × 90 km, see Fig. 5 for details) under RCP8.5. The left column shows the overview map from Lantmäteriet (The overview map, open data licence Creative Commons, (CC0), https://www.lantmateriet.se/en/) where green areas show forest areas while brown(ish) areas represent mountain tundra. The next three columns show simulated leaf-area index (LAI) of tree plant functional types (PFTs) averaged over three periods of the recent past and present century. The last column shows the fraction of the area for the three periods

Modelling of the vegetation change in the RCP8.5 scenario for the three biodiversity hotspot areas shows a strong decline of tundra areas, i.e. areas above the tree line, by the 2041–2060 period, and by 2081–2100, only fragmented tundra areas remain in the northernmost Abisko area (Fig. 6). By mid-century, the forest line advanced its areal extent quite slowly, but for Helags and Vindelfjäll the change was substantial towards the end of century, although not as strong as for tundra areas (Fig. 6). For the Abisko and Vindelfjäll areas, a successive upward elevational shift of all tree PFTs is projected (Supplementary Fig. S2a, b). In the Helags region, a similar trend is obtained, except that the mixed forest expands to lower elevations as temperate broad-leaved trees start to establish in the boreal forest (Supplementary Fig. S2c), i.e. the mixed forest class represents a boreal needle-leaved–mountain birch mixture in the beginning of the century and a needle-leaved–temperate broadleaf mixture in the later part with potential implications for biodiversity.

### Differences between scenarios and simulations

**Fig. 7:**
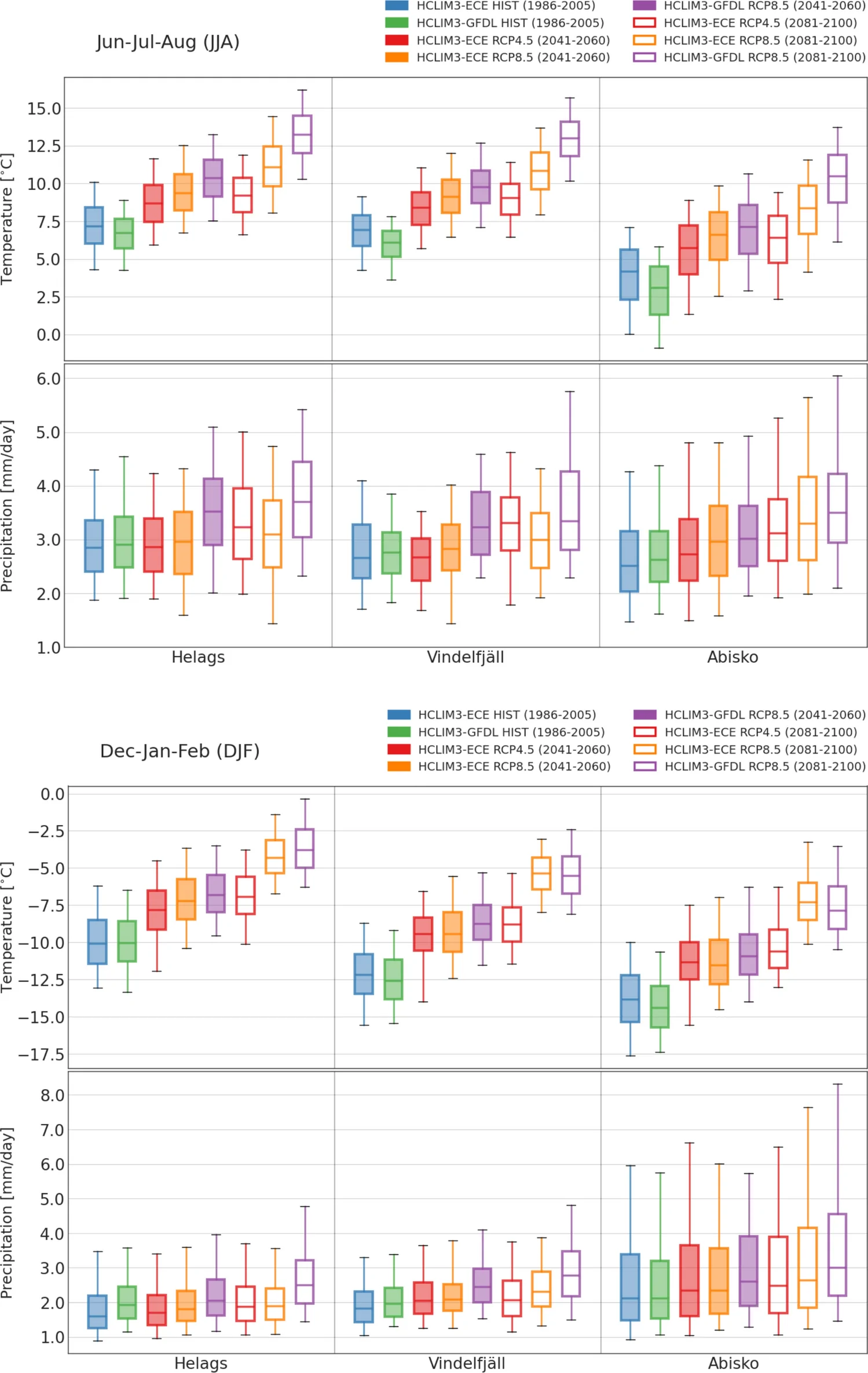
Climate projections with HCLIM3 driven by boundary conditions from the GCMs EC-Earth and GFDL under the scenarios RCP4.5 and RCP8.5. Seasonal mean temperature at two metres above ground (upper row) and precipitation (lower) for historical (1986–2005) and future (2041–2060 and 2081–2100) conditions as averaged over the regions shown in Fig. 6. The bar-whisker plots represent the 5th, 25th, 50th, 75th and 95th percentiles calculated from the interannual variability of the 20 years in each time period

All results presented above are derived from HCLIM downscaling the EC-Earth model under the RCP8.5 scenario except Fig. 2 where a comparison with HCLIM downscaling of GFDL is made. RCP8.5 has significantly higher greenhouse gas concentrations than RCP4.5 and consequently stronger forcing of the climate system. Notably, by the end of the century, the difference in effective radiative forcing is approximately 100%, whereas around mid-century the differences are smaller, 28% in 2050 (IPCC 2013). Here, we show differences between all three available HCLIM runs, including also GFDL as a driving GCM and an EC-Earth-driven simulation under the RCP4.5 scenario. Figure 7 shows twenty-year averages of mean summer and winter temperature and precipitation for the three biodiversity hotspot areas (Fig. 6). Also shown is the interannual variability of these meteorological variables (Fig. 7).

For both seasons, all three projections show increasing warming and an inclination of increasing precipitation with time. Generally, projected changes are larger for RCP8.5 than for RCP4.5 with larger differences for the end of the century than for mid-century. However, for mid-century in winter, the EC-Earth-driven RCP4.5 projection gives a stronger warming than the corresponding RCP8.5 projection both in Vindelfjäll and Abisko. Also, we note that, for summer at the end of the century, the EC-Earth-driven RCP4.5 projection gives a stronger increase in precipitation than the corresponding RCP8.5 projection both in Helags and Vindelfjäll. The figure also reveals differences depending on which GCM has been downscaled. In summer, it is clear that the GFDL-driven RCP8.5 projection shows stronger warming compared to the corresponding EC-Earth-driven projection, while in winter, differences are smaller and even reversed in two of the areas for the end of the century.

## Discussion

### Benefits of higher resolution

This study highlights the added value of using a higher spatial resolution when assessing projected changes in the climate conditions of the highly heterogeneous mountain landscape. Coarser resolutions lose significant details in climate variables, as demonstrated by the number of zero-crossings, rain-on-snow events and snow cover durations, all examples of climate indicators largely controlled by temperature conditions and associated with strong vertical and horizontal gradients in mountainous areas. High-resolution models, like the one applied here, also better represent variables with strong dependency on the horizontal variation. In addition to temperature, this includes near-surface winds, which are strongly linked to the orography of the landscape and where improvements over coarser-scale models have been shown in several studies (e.g. Jiménez and Dudhia 2013). Additionally, higher horizontal resolution with grid spacing of only a few kilometres allows models to run without parameterizations for deep convection, thereby more explicitly representing precipitation. Numerous studies have shown that convection-permitting models perform better not only for intense precipitation but also by reducing problems with excessive drizzle that is common in coarser-scale models (e.g. Lind et al. 2020). Furthermore, clouds, fluxes of radiation and near-surface relative humidity can be more realistically represented by high-resolution convection-permitting models (Lucas-Picher et al. 2021).

Our dynamic vegetation modelling also greatly benefited from increased spatial resolution. The modelled PFTs are typically distinguished by PFT-specific bioclimatic limits (e.g. often expressed as functions of growing degree-days and minimum or maximum temperatures) and PFT-specific physiological niches (e.g. temperature optima and sensitivities in process-based descriptions of photosynthesis) that depend on the climate inputs driving the models. This also applies to the modelled hydrological and biogeochemical processes, such as those relating to snowpack dynamics, soil water freezing and nitrogen cycling, which in turn influence vegetation dynamics through their effects on resource availability (Gustafson et al. 2021; Lagergren et al. 2024). The more detailed representation of the geographical distribution of environmental change will provide a stronger knowledge basis for different actors in the assessments of climate change consequences and adaptation strategies, while also considering the concurrent influence of land use, such as reindeer grazing. Higher resolution inputs to the dynamic vegetation model allow for more fine-grained simulations of tree- and forest lines. For example, rather than inputs of average daily temperature over a 12 × 12 km area, running the model in each of the sixteen 3 × 3 km cells with climate inputs that better reflect the topography of the region resolves vegetation on valleys and slopes that would not be captured otherwise. This aligns with earlier work for a single catchment at much higher resolution (Gustafson et al. 2021). 

A further dimension of resolution is the timescale used. Applying higher temporal resolution than that of the present study (daily) could provide additional relevant information, e.g. for decisions in reindeer herding, since short-term weather events may represent significant risks in its management (Rasmus et al. 2022; Rosqvist et al. 2022). More detailed studies of short-term weather events represent a possibility to further develop the work presented in this study.

### Environmental changes and their impacts

Our modelling results show the current trend in vegetation zone shifts, which aligns with the altitudinal advancement of shrubs and trees observed for the last decades (e.g. Kullman and Öberg 2009; Rundqvist et al. 2011; Hofgaard et al. 2013; Rees et al. 2020). An advancement of the tree line (70–90 m in elevation) has been observed in the southern Scandes over the last century, similar for pine, spruce and birch (Kullman and Öberg 2009). Our projections for the vegetation and tree line development until 2100 in a high greenhouse gas scenario (RCP8.5) align with other modelling studies (Hickler et al. 2012; Gustafson et al. 2021). These studies show that trees and forests will likely advance to altitudes they have not occupied since the warm period after the last deglaciation (Kullman and Öberg 2009) if greenhouse gas concentrations and temperatures continue to increase as strongly as in RCP8.5. Our results further indicate that large changes in vegetation are expected even under the more moderate RCP4.5 scenario. This becomes clear when comparing the projected climate change signals with those in the RCP8.5 scenario. In particular, temperature increases in the RCP4.5 scenario at the end of the century are close to those in the RCP8.5 scenario in the mid-century period, when significant changes in vegetation are projected. The results for the RCP8.5 scenario at the end of the century can serve as a proxy for a more distant future with continued accumulation of CO_2_ in the atmosphere, or for a situation with a significantly higher climate sensitivity than that in the driving GCM used here. A limitation of the simulated tree and forest line expansion is that a soil layer is always present in the model. The advancement of trees on former bare soil and rock and permanent snow/ice classes, e.g. as predicted for the Abisko area, is therefore probably overestimated, and parts of this area would likely become some type of shallow-soil associated tundra.

The projected loss of mountain tundra area (consistent with robust mid-century results for a smaller catchment arising from simulations using three driving GCMs for both RCP2.6 and RCP8.5 by Gustafson et al. (2021)) will limit an important food resource for reindeer. Warming experiments in the Arctic have found an increase in shrubs, in particular deciduous shrubs, and a decline in lichens in tundra communities (Elmendorf et al. 2012; Scharn et al. 2021). Since lichens and deciduous shrubs are important sources of food for reindeer (Eriksson et al. 2007), this shift in their abundance will likely drive changes in reindeer foraging. As well as summer grazing areas having an altered vegetation composition, a simulation of potential reindeer consumption in the Scandinavian Mountain Range showed a strong increase in summer grazing potential north of approximately 65.5°N towards the end of the century. However, close to the southern limit of the Swedish reindeer husbandry area, there will likely be a decrease (Lagergren et al. 2024), meaning that reindeer husbandry in the south will have to adapt to a more forest-bound livelihood in the expanding boreal forest.

Our predictions do not account for changes in lichens, as only a limited number of DVMs include lichens (Porada et al. 2013; Ma et al. 2024). Lichens are a crucial part of the reindeer’s diet during winter, as reindeer can smell lichens beneath the snow and use their curved hooves or antlers to access them (Heggberget et al. 2002). However, the projected increase in rain-on-snow events and zero temperature crossings, often poorly represented in DVMs (Pascual et al. 2024), creates ice layers on the ground surface and within the snowpack (Bokhorst et al. 2016). This phenomenon, already observed in northern Sweden (Rosqvist et al. 2022), can result in snow profiles with multiple ice layers reducing reindeer’s ability to locate and access lichens. Consequently, these conditions pose significant challenges for reindeer herding and may necessitate supplementary feeding (Harnesk 2022).

It has become increasingly clear that nature conservation must adapt to climate change and its ecological consequences to manage and protect specific conservation values in the long term (Huntley et al. 2018). This need is reflected in the most recent international conservation standards (Conservation Measures Partnership 2020). Our study shows a loss of conservation values in the tundra of the Scandinavian Mountain Range, based on the very substantial decline of genuine tundra areas projected by mid-century (likely consistent across emission scenarios) and especially by 2100 in the warmest scenario. High geographical resolution in the assessment of both climate and ecological change, as exemplified in our study, is crucial for considering climate change in nature conservation. Ecosystem types with different degrees of vulnerability are intermixed on a local scale, and the relationship between climate change and microclimate must be considered to understand the ecological consequences for plants and other organisms (Huntley et al. 2018; De Fenne et al. 2021). A decline in lichens and bryophytes has already been observed at mountain tops in northern Sweden (Hagenberg et al. 2022). The projected loss of mountain tundra in all three biodiversity hotspot areas in our study is very pronounced and will lead to both habitat and species loss.

Our study projected substantial shortening of the snow season under the RCP4.5 (not shown) and RCP8.5 scenarios, which could significantly affect winter tourism. European ski tourism is facing important challenges from climate change (Rutty et al. 2021). These challenges include not only a shortened season but also increasing costs for snowmaking (Berard-Chenu et al. 2021; François et al. 2023), which will require additional energy and increase the carbon footprint of the activity. However, northern Sweden may suffer less from climate change than areas further south in Sweden and Europe (Rice et al. 2022; François et al. 2023). To adapt to and plan for the changing climate, the tourist industry will benefit from services providing relevant information on observations and projections for future risks and opportunities (Rutty et al. 2021). High-resolution climate modelling of relevant indicators (Abegg et al. 2021) can play an important role in this adaptation. While much of the discussion of climate change impacts on tourism has focussed on winter and snow activities, it is also important to consider the climate change-induced advancement of the forest line, highlighted in this study and its effect on the appearance of the landscape. This will have profound effects on both summer and winter tourism and their distribution in the mountains. Weather extremes, such as heavy rainfall events, and security aspects related to changing frequency and geographical distribution of avalanches and landslides under climate change, can also affect tourism (Laute et al. 2018; Jemec Auflič et al. 2023). Taken together, this will likely result in a concentration of mountain tourists in the remaining genuine tundra environments, which could increase disturbance of wildlife and reindeer herding in these areas, as well as an increased vulnerability to natural disasters for tourists.

There are additional climate-related indicators that may be relevant to reindeer herding that were not included in this study. Exceptionally deep snow is one such factor. A key challenge in modelling this aspect is the lack of snow depth data that accurately captures extreme conditions at the required level of geographical detail across large areas. Consequently, calibration and validation of this component are currently difficult to achieve. A further variable of potential interest is the development of wind speeds with possible implications for, e.g. snowpack dynamics. Although strong indications of a systematic change in the large-scale wind climate in the region under consideration are missing in simulations of future warmer climate conditions, wind patterns may still change due to local thermal changes. The high-resolution HCLIM model used here has been shown to have an improved representation of wind speed distribution in the landscape, not only for large-scale driven windy conditions but also for conditions governed by local thermal contrasts (Belušić and Lind 2025).

### Limitations arising from uncertainties in simulations and modelling setup

Our use of different driving GCMs impacts the results, as does the choice of RCP scenario. By mid-century, the differences between the two scenarios and the different driving GCMs are however moderate, implying that the projected changes in vegetation are relatively robust until then.

We note that scenarios with lower concentrations of greenhouse gases, such as RCP2.6, could result in a weaker response also for the mid-century timeframe. In a longer time perspective, the results become more sensitive to different scenarios and how strongly the climate system responds to them. This is consistent with the higher (50 m) resolution LPJ-GUESS projections made by Gustafson et al. (2021) for the Lake Torneträsk sub-arctic landscape in northern Sweden, in which the model was driven with downscaled RCP2.6 and RCP8.5 climate scenarios from three CMIP5 GCMs selected specifically to capture the spread in the modelled mean annual temperature for 2071–2100. Simulated average tree line elevation rose in mid-century, but did not differ greatly across GCMs and RCPs, with robust differences between RCPs only emerging at the end of the present century.

A further source of variability in modelling results is related to uncertainty in a model’s parameter values, which could significantly impact on the results (Wramneby et al. 2008; Russo et al. 2020). Model comparison studies (Xia et al. 2017; Coppola et al. 2020) can assess these types of uncertainties. Factorial experiments, where drivers are changed one at a time, can provide further insight into the key drivers of modelled dynamics, e.g. where Gustafson et al. (2021) highlighted the importance of nitrogen availability for tree line advance. However, accounting for all these aspects is beyond the scope of this study. To capture in full future uncertainties in vegetation change (or the sensitivity of key metrics such as average tree line altitude to climate change) would require simulations with multiple DVMs, each considering its own parameter uncertainty, forced with multiple climate change datasets arising from differences in model structure (i.e. which GCM(s) to use), scenario (i.e. which emission scenario to follow) and internal model variability (i.e. which GCM ensemble member(s) to use). Comparing with a large ensemble of GCM simulations, Lind et al. (2023) noted that of the two RCP8.5 simulations downscaled with HCLIM here, the projected climate change signal in EC-Earth is close to the median for both temperature and precipitation in a wider north European area. Contrastingly, GFDL is among the GCMs with the strongest increase in both temperature and precipitation. This implies that the current setup captures a substantial part of the full range of GCM projected climate change.

Uncertainty may arise from downscaling method (i.e. choice of regional climate model(s), RCM(s), to use). Therefore, the presented results should be viewed as an example of a novel high-resolution impact modelling chain and framework over a large and ecologically important region.

For Europe, the coordinated regional climate downscaling experiment (EURO-CORDEX) provides a large ensemble of GCM-RCM combinations that can be used to further address this uncertainty at 12 km horizontal resolution (Jacob et al. 2020). On a global scale, coordinated efforts such as The Inter-Sectoral Impact Model Intercomparison Project (ISIMIP) design impact model simulation protocols to capture some of these uncertainties, but typically at much coarser resolutions than used here (Rosenzweig et al. 2017).

In addition to the need for studies where multiple DVMs are driven at high resolutions to explore uncertainty in impacts for a wider range of climate scenarios, several developments in process and vegetation descriptions in DVMs would increase their utility for impact studies and the provision of ecosystem service indicators. Including mechanistic and ecologically realistic non-vascular functional types, such as lichens and bryophytes (Porada et al. 2023), would not only improve the projected impacts on areas suitable for reindeer foraging but also improve the physical description of soil temperature and permafrost change (Porada et al. 2016) through their effect on the thermal and hydraulic properties of the land surface. Climate-related disturbances, such as winter warming events (Pascual et al. 2024) and the development of wildfires (Emmet et al. 2021), also require further developments to improve understanding of their impact on high-latitude and altitude ecosystems, reindeer foraging and the thermal development of the soil and snowpack. Finally, the dynamic and coupled evolution of regional climate and terrestrial ecosystems is not yet standard in regional climate models at the resolutions considered here. Associated biophysical feedbacks are expected to alter local and seasonal air temperatures with a magnitude that is significant compared to the background climate warming signals (e.g. Wramneby et al. 2010; Zhang et al. 2018). Therefore, efforts should be made to include these interactions for more coherent and consistent regional climate projections.

## Conclusions

We conclude that climate change will result in major changes concerning important, stakeholder-relevant indicators of ecological change in the Scandinavian Mountain range. These changes include shorter snow seasons, more frequent rain-on-snow events except in summer and an increasing risk of internal ice layers within and below the snowpack, impacting reindeer grazing possibilities and the livelihood of both reindeer herding communities and tourism. The upward shift in both tree and forest lines, and the resulting reduction of tundra ecotones is consistent with earlier studies using a broader range of climate inputs and is likely to pose threats to species and ecosystem survival, influencing land use and local livelihoods. Secondly, the higher spatial resolution of the model analysis used in this study is instrumental in accurately projecting ecological change in mountainous terrain. Research and society need to engage with the challenges that reindeer herding, tourism and nature conservation will face over coming decades with respect to the physical and ecological transformations that will follow climate change. We have modelled the direction of ecological change resulting from ongoing climate change, and for the next few decades, the projected change is robust, independent of climate projections and different RCP scenarios. For the second half of the century, the rate of change will depend on the extent to which greenhouse gas emissions are reduced.

## Supplementary Information

Below is the link to the electronic supplementary material.

Supplementary file1 (PDF 780 KB)

## Data Availability

Selected data (climate, air pollution and ecosystem development) is available in Zenodo: https://doi.org/10.5281/zenodo.7573171. LPJ-GUESS model results are available at DataGURU: https://doi.org/10.18161/j395-1j66.

## Footnotes

1
https://www.smhi.se/forskning/forskningsprojekt/vara-forskningsprojekt/biodiv-support----scenariobaserat-beslutsstod.

2
https://surfobs.climate.copernicus.eu/dataaccess/access_ngcd.php.

## Code availability

The LPJ-GUESS code used in the presented simulations is published at Zenodo: https://doi.org/10.5281/zenodo.8262590.
