# Supplementary material for: Highly resolved projections of climate and vegetation indicate major changes in northern Scandinavia: Implications for reindeer herding, tourism and nature conservation
Source: Ambio. 2026 May 22;55(12):2961–79. doi: 10.1007/s13280-026-02423-w (PMC13731605; doi:10.1007/s13280-026-02423-w)
Supplement: Supplementary file 1 [file 13280_2026_2423_MOESM1_ESM.pdf]

Supplementary information for Ambio paper:

Highly resolved projections of climate and vegetation indicate major changes in northern Scandinavia – implications for reindeer herding, tourism and nature conservation

Håkan Pleijel, Fredrik Lagergren, Erik Kjellström, Petter Lind, Paul A. Miller, Robert G. Björk, Tinja Olenius, Danijel Belušić, Mats P. Björkman, David Lindstedt, Gunhild Rosqvist, Camilla Andersson

Corresponding author: Håkan Pleijel, University of Gothenburg, Department of Biological and Environmental Sciences, P.O. Box 463, SE-40530 Göteborg, Sweden

Telephone: +46733100700

E-mail: hakan.pleijel@bioenv.gu.se

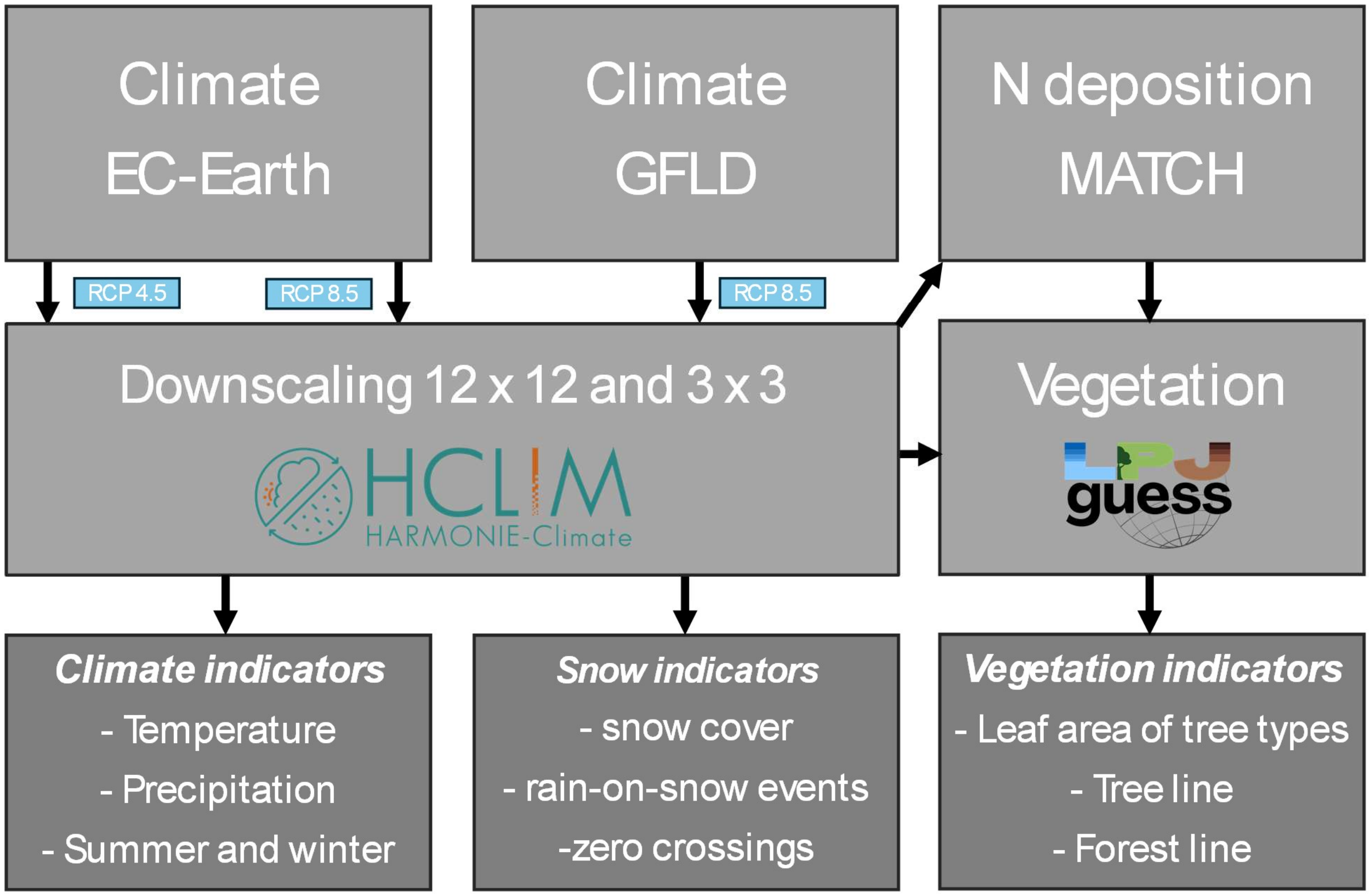

Figure S1. Schematic overview of the modelling tools (light grey) used and combined (arrows) to project the development of the societally relevant indicators identified and investigated (dark grey). The representative concentration pathways (RCPs), i.e. greenhouse gas emission scenarios used in different parts of the analysis, are also presented (small blue rectangles).

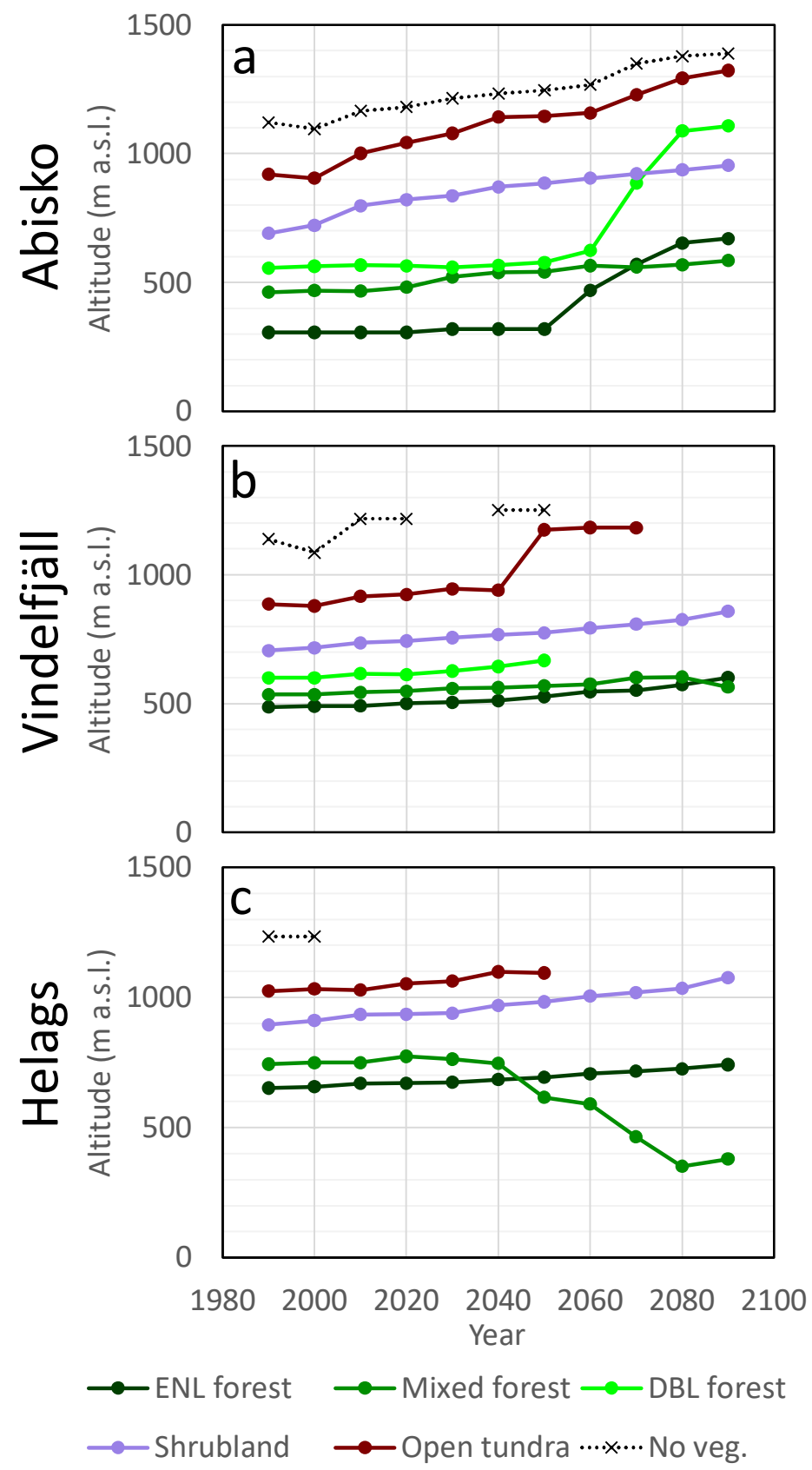

Figure S2. *Development of the average altitude of grid cells simulated to belong to a certain vegetation type (ENL is evergreen needleleaf and DBL is deciduous broadleaved) within the three mountain biodiversity hotspot areas (a-c) under RCP8.5. The vegetation types are based on the ten-year average LAI of the different plant functional types belonging to each type shown here. In Vindelfjäll the "No veg." class is missing in 2030 but reappears in 2040 for one climate cell, which is related to the random disturbances and climate in single years.*
